# Changes in the perception of upright body orientation with age

**DOI:** 10.1371/journal.pone.0233160

**Published:** 2020-05-29

**Authors:** Sophia Nestmann, Hans-Otto Karnath, Heinrich H. Bülthoff, Ksander Nikolas de Winkel

**Affiliations:** 1 Division of Neuropsychology, Centre of Neurology, Hertie-Institute for Clinical Brain Research, University of Tübingen, Tübingen, Germany; 2 Department of Psychology, University of South Carolina, Columbia, South Carolina, United States of America; 3 Max Planck Institute for Biological Cybernetics, Tübingen, Germany; Universite Toulouse III - Paul Sabatier, FRANCE

## Abstract

To determine own upright body orientation the brain creates a sense of verticality by a combination of multisensory inputs. To test whether this process is affected by aging, we placed younger and older adults on a motion platform and systematically tilted the orientation of their visual surroundings by using an augmented reality setup. In a series of trials, participants adjusted the orientation of the platform until they perceived themselves to be upright. Tilting the visual scene around the roll axis induced a bias in subjective postural vertical determination in the direction of scene tilt in both groups. In the group of older participants, however, the observed peak bias was larger and occurred at larger visual tilt angles. This indicates that the susceptibility to visually induced biases increases with age, possibly caused by a reduced reliability of sensory information.

## Introduction

To act purposeful in the environment, we evaluate the orientation of objects surrounding us as well as the orientation of our own bodies in space. To determine whether an object or our bodies are in line with the direction of gravity, the brain is assumed to rely on a sense of verticality. This percept is thought to be based on a combination of visual information, proprioceptive feedback, graviceptive information from the vestibular system and specialized graviceptors located in the trunk [1], as well as the prior knowledge [2] that gravity typically aligns with the body’s longitudinal axis. Different theories exist on how this information is combined, but theories typically agree that information that is inferred to reflect verticality is integrated as a weighted average, with weights inversely proportional to sensory noise of the respective modality [2–5].

Throughout life, the acuity of the different sensory modalities that provide verticality information changes, which raises the question on how verticality perception might change with age. Significant age-related decline of the vestibular system has frequently been reported [6–8]. Bermúdez et al. [9], for example, report increased thresholds for the detection of tilts in either direction, including roll tilt, for older participants. It is likely that vestibular noise increases with age, which might cause a decrease of the contribution of vestibular information to verticality perception [10]. Consequently, older participants may be more susceptible to large visually induced biases in situations of uncertainty.

Experimentally, this can be tested by separately manipulating visual and inertial cues in an ‘Alternative Reality’ (AR) setup, as was done by de Winkel et al. [5]. To investigate how the interplay between different inputs on verticality estimation changes with age, we tested a group of younger participants as well as a group of older participants. We adopted the AR paradigm by de Winkel et al. [5] to manipulate the orientation of the participants’ actual visual environment in real-time and thus create various (in)congruent visual-physical roll tilt stimuli. Verticality perception was assessed by the subjective postural vertical (SPV): participants were seated on a motion platform while wearing the AR system. Using this setup, we independently manipulated physical and visual roll-tilt in a series of experimental trials. For each trial, participants were asked to adjust the platform − and thus the orientation of their own body − until they again perceived it as being ‘upright’.

We predicted that for older participants an increased noise in the vestibular system would lead to relatively larger weights for visual information, ultimately resulting in larger SPV biases compared to younger participants.

## Methods

### Setup

**Fig 1:**
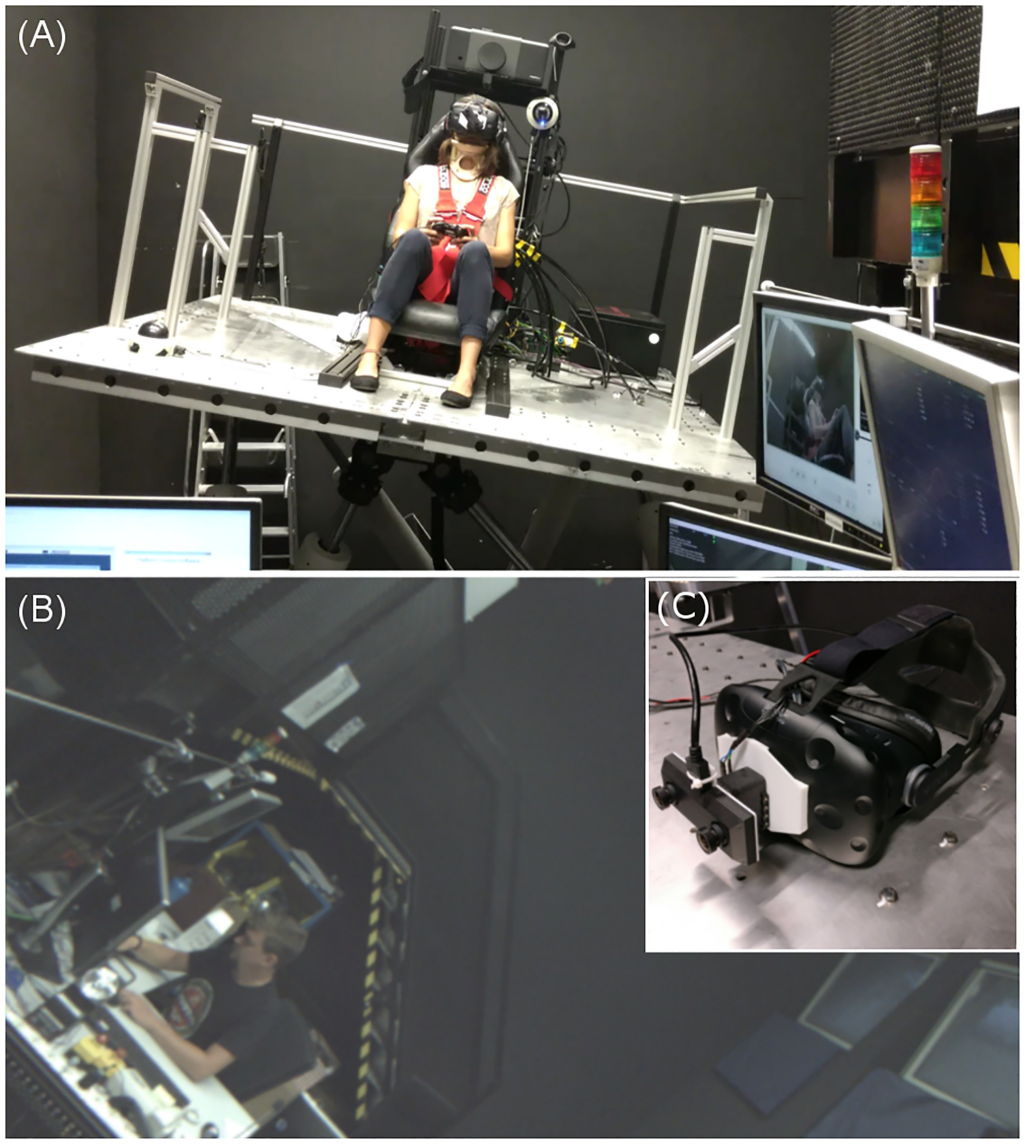
Experimental setup. (A) Participants sat on a motion platform. They could control their roll tilt angle relative to gravity by moving the platform via an Xbox Controller. (B) Monoscopic screenshot of participant’s view. Participants saw a statically tilted version of their real time immediate surrounding. (C) Visual stimuli were presented using a head mounted display (HMD) with a connected camera. Fig 1c) is taken from de Winkel et al. [5] with the authors’ permission.

The setup of the experiment was identical to the one described in de Winkel et al. [5] and the same devices were used in the experiment. Participants were seated in a bucket seat (RECARO GmbH, Stuttgart, Germany), which was installed on a motion platform (eMotion 1500 hexapod motion system, Bosch Rexroth AG, Lohr am Main, Germany; Fig 1a). By using a head mounted display (HMD) with a connected stereo camera (Fig 1c), participants were presented tilted versions of their immediate surrounding in a real-time AR setup (Fig 1b). Facing the entrance and control area of the experimental room, participants were presented an image rich in verticality cues. Polarity information from objects as well as slight movements of the experimenter provided natural indicators on verticality.

The image captured by the stereo camera (OVR Vision Pro stereo camera, Wizapply, Osaka, Japan) was presented stereoscopically on the screens of the head mounted display (HTC Vive, New Taipei City, Taiwan) at a rate of 45 frames per second. The screens’ resolution was 1080 × 1200 px, with a field of view of 100° × 110°. Different tilt angles of the visual input were realized by rotating the camera, which was mounted on the HMD, around the participants’ naso-occipital axis with a Dynamixel AX-12Aservo motor (Robotis, Lake Forest, California, United States).

A 5-point safety harness (SCHROTH Safety Products GmbH, Arnsberg, Germany) was used to secure participants in the seat. Using the motion platform, participants could be tilted to approximately ±13° around the naso-occipital axis. By asking participants to tighten the seat belt as much as possible without feeling uncomfortable, somatosensory feedback in terms of pressure information from the seat was kept approximately constant. To prevent visual feedback on platform orientation relative to the room, the seat was moved close to the edge of the platform and participants were instructed to sit as upright as possible, so that they were not able to see either the platform or their legs’ orientation relative to the room. To both prevent unintentional corrective head-tilts in response to scene tilt, as well as passive head tilts evoked by platform movement, participants wore a cervical collar. It was fitted tightly for each individual participant, in the same manner for younger and older participants. It should be noted that cervical collars are not infinitely stiff, and that there may have been minor residual reflexive head tilts. Because of their compensatory nature, such tilts potentially reduce the effects of visual tilt stimuli. To avoid that sounds created by the motor of either platform or camera would be used as a spatial reference, participants wore earplugs with a 33dB signal-to-noise ratio (Honeywell Safety Products, Roissy, France) as well as active noise cancelling headphones (Plantronics, Santa Cruz, California, United States) that played white noise during platform and camera rotations. Participants could adjust the roll-tilt angle of the platform using the left (clockwise from an observers perspective) and right (counterclockwise) trigger buttons on an Xbox Controller (Microsoft, Redmond, WA, United States). Each button press moved the platform by ±0.05°. Sustained button pressed induced a smooth motion as the signal was passed through an integrator and a low-pass filter. Platform movement started immediately at button press. The experimental setup was controlled using Simulink software (The MathWorks, Inc., Natick, Massachusetts, United States).

### Task & stimuli

Verticality perception has been operationalized in different ways. Prominent measures are the Subjective Visual Vertical (SVV), the Subjective Haptic Vertical (SHV), and the Subjective Postural Vertical (SPV). The SVV is usually measured by asking participants to align a visual object such as a bar with the subjective experience of upright. It is thought to reflect the perceived orientation of the head relative to gravity. The SHV is measured by having participants manually align a physical rod with gravity and is thought to reflect the perceived orientation of the body. The SPV similarly represents the orientation of the body in space, but involves methods that allow participants to adjust the orientation of their entire body. In contrast to the SHV, the SPV thus does not require a transformation of an internal representation of body orientation relative to gravity into an external adjustment of rod orientation. While all of these methods measure a form of verticality perception, and healthy individuals perform quite precisely for either approach [11–14], brain damaged patients can exhibit selective disturbances of either SPV or SVV [11,15–17]. This suggests that different representations of verticality exist, that are constructed by different subsystems. In the present experiment, we aimed at measuring the perception of body orientation in relation to gravity, which, in our opinion, is most directly and appropriately measured by the SPV.

SPV was measured by instructing participants to adjust the platform tilt until they perceive themselves to be oriented upright in space. The task was performed by first moving the platform to initial conditions, specified by physical tilt angles S_I_ = {-10°, -5°, 5°, 10°} in combination with a visual tilt angle S_V_ (see below), and subsequently allowing participants to reorient the platform using the buttons of an Xbox Controller. In each trial, participants confirmed the position for which they perceived themselves as upright by button press. After this, they started the next trial by a second button press. Platform and camera tilt were manipulated separately; within each trial, camera tilt was kept at a constant target angle relative to gravity by correcting for platform movements. Note that S_I_ angles only represent starting positions of platform adjustment. Breaks were offered every 28 trials with additional breaks if desired. Since no time limit for platform adjustment was set, the duration of each trial depended on participants’ pace. On average, the task took about 30 minutes on the platform.

The angles of camera tilt presented to the participants were S_V_ = {-36°, -13°, -5°, 0°, 5°, 13°, 36°}; each possible combination of S_I_ and S_V_ conditions was presented four times, resulting in 112 trials. In the subgroup of younger participants, we furthermore included three participants who were presented tilt angles of S_V_ = {−36°, −13°, 0°, 13°, 36°}. For these participants, each condition was presented five times (100 trials). For both platform and camera, negative tilt angles indicate counterclockwise tilt from an observer’s perspective. For camera tilt, counterclockwise tilt translates to a perceived clockwise tilt of scene.

### Participants

We recruited 27 volunteers for the experiment of whom one participant could not complete the experiment due to motion sickness. One participant during debriefing admitted to have closed his eyes during the experiment to be better able to judge verticality. As this conflicts with explicit task instructions, we excluded his data from the analysis. One participant answered unsystematically during the experiment, not reflecting on the position of upright in the different conditions. Verification of data quality revealed a large variance in the responses of this participant. We calculated average variance values for each possible combination of S_I_ and S_V_. Overall, participants showed significantly less variance in their data than this participant (t = -132.28, p <0.001). This supported our observation of unsystematic responses and led us to exclude this subject. An additional 12 participants performed the experiment with a larger range of visual tilt stimuli. The results of these experiments are not included here.

Data of 24 participants were included in the final analysis. We tested 16 younger participants (mean age: 27.6 years, range 21–44 years, female: 10) and eight older participants (mean age: 67.9, female: 4). Characteristics of the samples can be inspected in Table 1. Participants were financially compensated for their participation at a rate of €8 per hour. All participants gave written informed consent before participation. The individuals, who can be seen in Fig 1 of this manuscript have given written informed consent (as outlined in PLOS consent form) to publish these images.

**Table 1:** Subgroup characteristics.

Subgroup	Group size	Mean age (SD)	Gender	S_V_
Younger Participants	3	27.33 (3.09)	female: 1, male: 2	{-36°, -13°, 0°, 13°, 36°}
	13	27.62 (5.58)	female: 9, male: 4	{-36°, -13°, -5°, 0°, 5°, 13°, 36°}
Older Participants	8	67.88 (6.51)	female: 4, male: 4	{-36°, -13°, -5°, 0°, 5°, 13°, 36°}

The table presents the size of each subgroup, mean age and standard deviations (SD), as well as gender of the participants and the presented visual tilt angles S_V_.

### Ethics statement

The experiment was conducted in accordance with the declaration of Helsinki and was approved by the ethical committee of the medical faculty of the Eberhard Karls Universität Tübingen, Germany, reference number 352/2017BO2.

### Data analysis

From the raw data collected for the 24 subjects, 112 trials were removed because of technical issues (failure of headphones or camera) or because participants accidently answered by button press that they perceived the current position as upright, instead of moving the platform with another button. Whereas some participants indicated these trials immediately, others only reported to have accidently given the wrong answer in some trials during debriefing, so that the corresponding trials had to be identified from the data afterwards. We excluded these trials on a conservative level to avoid that they artificially increased the observed bias: responses were considered accidental if the starting position S_I_ was identical to the participant’s response and differed more than ±2.5 degrees from the response given in the preceding trial. This approach took into consideration that participants might have actually felt upright at the presented S_I_. In addition, outliers were excluded on the individual level by identifying responses differing more than ±3 standard deviations (SD) from the individual mean (18 trials). After excluding trials due to technical problems, wrong button presses, and outliers, 95% of the initial 2640 trials remained in the analysis.

To account for the possibility that participants might not have been seated perfectly upright and to exclude this potential bias from upright estimates, we subtracted each participant’s mean answer at S_V_ = 0° from each given answer. This centered each participant’s data to an individual baseline making the data comparable between individuals. To assess whether there were differences in the relationship between visual input manipulation and the observed bias in SPV between the two age groups, we fitted linear mixed effect models to the data using the lme function provided by R’s nlme package [18,19]. Random intercepts and slopes were estimated for the variable *participant*, to account for repeated measurements. A random intercept was estimated for the variable S_I_ to consider the effect of different starting angles of the platform. The variables S_V_ and *group* were added as fixed effects to assess the differences between groups and tilt angles. Furthermore, we included the interaction of S_V_ and *group*. An interaction effect would translate into an increased or decreased effect of visual tilt on the bias observed in SPV estimates for one subgroup. Heteroscedasticity was accounted for by allowing different variances in the subgroups, identified by the varIdent function provided by R’s nlme package [18,19]. The model was fit using restricted maximum likelihood (REML) estimation. Statistical significance of parameter estimates was determined using the anova function provided by R’s nlme package [18,19]. As we did not expect an effect of visual tilt direction (clockwise vs. counterclockwise), we collapsed the data over positive and negative angles, by changing the sign of visual tilt angles and responses for all visual tilt angles smaller than 0. Formally, the collapsing procedure can be described as

$$response_{collapsed}(S_{V})=\{\begin{array}{ll} response(S_{V})*(-1), & for\;S_{V}<0\\ response(S_{V}), & for\;S_{V}\geq 0 \end{array},$$

and

$$S_{V\_abs}=\left|S_{V}\right|.$$

The precise equation used for the analysis was

$$response_{collapsed}\sim -1+S_{V\_abs}*group+(1+S_{V\_abs}\left|Participant)+(1\right|S_{l}).$$

## Results

**Fig 2:**
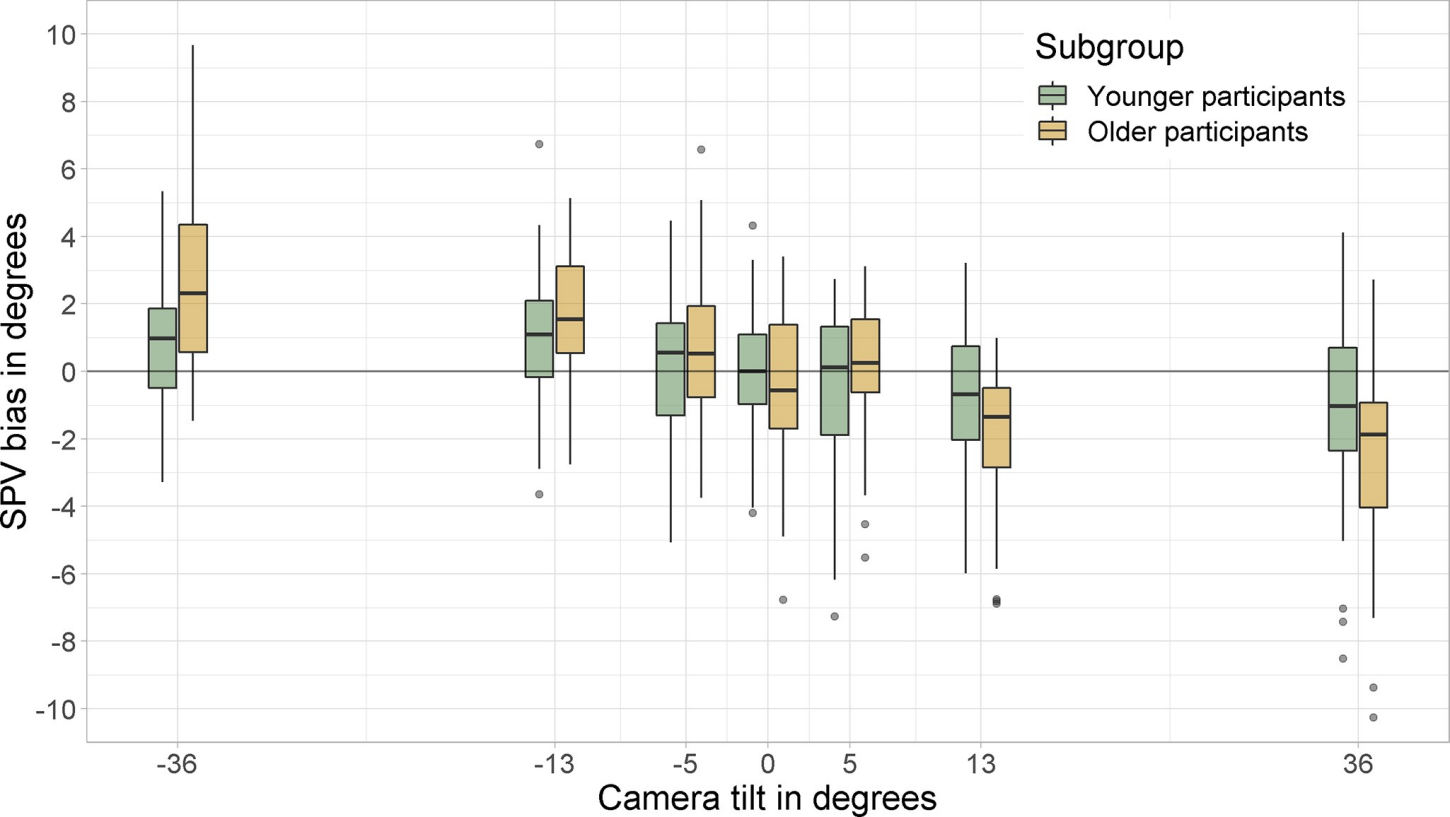
Raw data of participants’ SPV estimates from the different groups. The amount of visual tilt is presented on the x-axis, participants responses on the y-axis. Green colour represents the data of younger participants. Data of older participants is presented in yellow. Positive values indicate clockwise tilt of camera and platform. Note that clockwise camera tilt translates to a counterclockwise percept of scene tilt. Camera tilt seems to induce a bias of SPV perception in the direction of scene tilt for all subgroups. The data furthermore suggests that largest biases are observed for the subgroup of older participants.

Fig 2 shows participants’ responses (raw data). Camera tilt seems to induce a bias of SPV perception in the direction of scene tilt for both groups. The data suggests that larger biases can be observed for the subgroup of older participants. S1 Fig furthermore displays SPV judgments of each individual participant separately.

Statistical analysis revealed a significant main effect of the factor S_V_ (F = 15.833, p<0.001) and a significant interaction of S_V_ and *group* (F = 2.976, p = 0.031). Parameter estimates with the subgroup of younger participants set as reference group are summarized in Table 2. Post hoc tests with p-value adjustment according to Tukey’s method revealed that for the factor *S*_*V*_, there was a significant difference in bias for the contrasts 0° and |13°| visual tilt (t = -6.162, p<0.001), 0° and |36°| visual tilt (t = 4.920, p<0.001), |5°| and |13°| visual tilt (t = 5.984, p<0.001) as well as for the contrast of |5°| and |36°| visual tilt (t = -4.710, p<0.001).

**Table 2:** Parameter estimates.

Model Component	ß	SD	df	t	p
0° tilt	-0.00328	0.12623	2408	-0.026	0.979
5° tilt	-0.27929	0.12341	2408	-2.263	0.024
13° tilt	-0.84259	0.18044	2408	-4.670	<0.001
36° tilt	-0.99098	0.41771	2408	-2.372	0.018
Group	0.00355	0.30017	23	0.012	0.991
5° tilt x Group	-0.14290	0.37855	2408	-0.377	0.706
13° tilt x Group	-1.02016	0.43797	2408	-2.329	0.020
36° tilt x Group	-1.82128	0.77172	2408	-2.360	0.018

The group of younger participants was set as reference group. The table displays estimated coefficients for each model term, the corresponding standard deviations (SD), degrees of freedom (df), as well as t-statistics and p-values for each term. For interpretation of the coefficients, note that clockwise camera tilt translates into counterclockwise perception of visual tilt and thus negative weights indicate tilt in scene direction.

**Fig 3:**
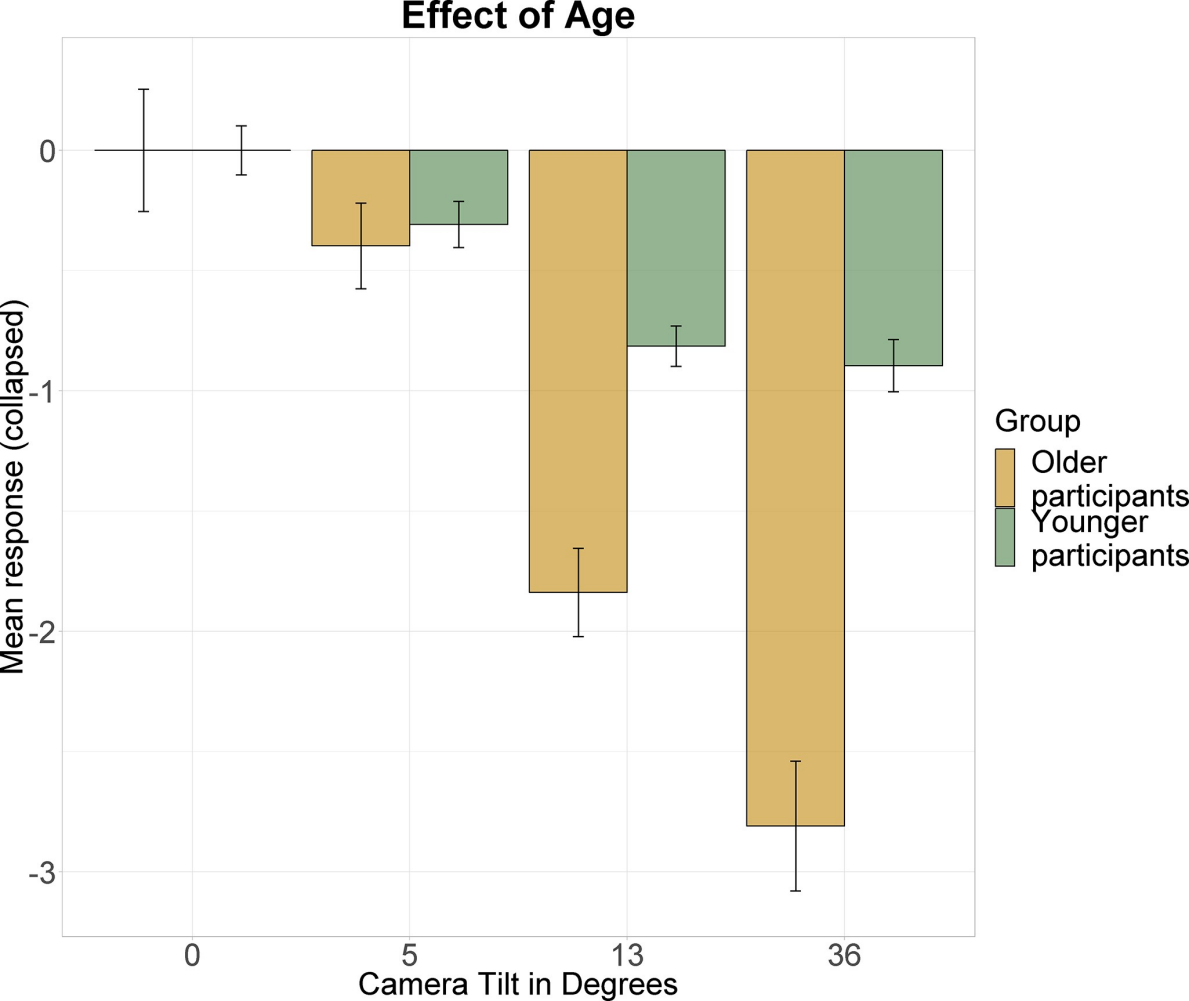
Mean SPV estimates with error bars indicating ±1 standard error. Data was collapsed as no tilt side specific result was expected. The amount of visual tilt is presented on the x-axis, participants’ collapsed responses on the y-axis. Green colour represents the data of younger participants, data of older participants is presented in yellow. Camera tilt induces a bias of SPV perception in the direction of scene tilt for both subgroups. The largest biases are observed for the group of older participants.

Post hoc tests concerning the interaction term revealed significant differences in SPV bias for visual tilts of 0° and |13°| both in the subgroup of younger (t = 4.041,p = 0.001) and older participants (t = 4.823,p<0.001). Also, both younger (t = 3.533, p = 0.009) and older participants (t = 4.892, p<0.001) produced significantly different estimates in the conditions |5°| and |13°| visual tilt. The contrast of 0° and |36|° visual tilt was only found to be significant in older participants (t = 4.346, p<0.001), as well as the contrast of |5°| and |36°| visual tilt (t = 4.357, p<0.001). Significant differences were furthermore found between 0° tilt in younger and |13°| in older participants (t = 5.837, p<0.001), 0° tilt in younger and |36|° in older participants (t = 4.523,p = 0.003), |5°| tilt in younger and |13°| in older participants (t = 4.986, p = 0.001), as well as |5°| tilt in younger and |36°| in older participants (t = 4.082, p = 0.009). Mean responses for the different tilt angles and groups are shown in Fig 3.

## Discussion

In the current study, we investigated the relationship between tilted visual input and perceived upright body posture in groups of younger (mean age: 27.6 years) and older (mean age: 67.9 years) participants, measuring subjective upright body orientation (SPV). In both age groups, the data indicates that tilting the visual scene around the roll axis leads to bias in SPV determination, in the direction of scene tilt. This is consistent with results of previous studies [20–23].

The size of SPV bias, which we observed in our experiment, can be evaluated regarding experiments using similar approaches. Isableu et al. [21] tested participants’ body orientation while standing in the sharpened Romberg position and being presented a tilted frame. While the pattern of bias in the direction of scene tilt is the same as observed by us, the reported mean biases are smaller (below one degree). Furthermore, Bringoux et al. [23] described the orientation of head position during exposure to a tilted frame. Head orientations occurred in the direction of frame tilt and were also smaller than the biases observed in our experiment (around one degree). A possible explanation for the differences between these and the present findings is that efference copies in standing posture may have limited the amount of bias compared to the present task. We also showed participants their actual environment and thus a scene much richer in visual information, which has been shown to be more compelling [24].

Our analysis demonstrated that older participants produce larger errors in verticality estimates evoked by visual tilt, most prominent at larger tilt angles. This is in line with results of Poulain et al. [25], who described that older individuals, compared to younger participants, show a larger body tilt when being presented a tilted visual frame. In a rod and frame task, Alberts et al. [10] tested participants of different age groups and observed that presenting a tilted visual frame leads to a bias in verticality estimation which increases with age. By fitting a Bayesian optimal integration model they showed that this is caused by sensory reweighting. More precisely, as vestibular information becomes less reliable with age, visual information receives increased weight during the determination of verticality. These results are very similar to ours, although stimuli were less realistic than those used in our experiment and verticality was measured using the SVV instead of the SPV. Research on postural control suggests that humans update body posture by using input from different sensory modalities. Information gained from these sub-systems is not always equally reliable or useful. It is thus important, that the brain dynamically adapts the weights attributed to the different sensory modalities to account for possible input alterations [26]. Park et al. [7] reported the steepest reduction in the number of vestibular ganglion neurons in the temporal bone to happen between 30 and 60 years of age. This means that the two age groups investigated in our experiment represent both a sample of participants before and after significant vestibular decline. Taken together, these findings support the idea that larger visual biases for older participants observed in our experiment might be a consequence of an age-related reduction of input reliability of the non-visual sub-systems, such as the vestibular system, relative to the visual system. A higher susceptibility to input from the visual modality observed in older participants might thus be a consequence of reduced weights assigned to vestibular information and increased weight assigned to visual information during verticality determination [10,27,28].

The observations of our experiment can furthermore be looked at in terms of a Causal Inference Model (CI model) on verticality perception, as described by de Winkel et al. [5]. The authors suggest that the brain creates the percept of upright based on causal inference; proposing that multisensory information on verticality is integrated, as in the model by Alberts et al. [10], only when it is likely to have a common cause, and segregated otherwise. Basically, the model reflects the idea that in cases of large discrepancies in multi-sensory information on verticality (in our experiment induced by the alternative reality approach), the brain may consider it unlikely that the visual information reflects the own posture by comparison to inertial information. Aging might affect the brain’s tolerance for discrepancies of this kind. To be able to interpret our results in terms of the CI model, we conducted an additional analysis. As for the present experiment it is not straightforward to fit the CI model directly to the data, we chose to approximate it by fitting a polynomial including a first degree and a third degree term. Specifically, we predicted that SPV estimates would be biased by visual tilt, but that this bias would gradually reduce as visual tilt angles become more extreme. The details of the data analysis are given in the S1 Data. The results show that, independent from age, less weight is given to visual information at largest tilt angles. This observation is compatible with predictions of the CI model of verticality perception [5] (for comparisons between and considerations on the CI model and alternatives, see [29–31]. However, the CI model on verticality perception was based on observations of SHV estimation [5], whereas we used SPV as a more direct measure of verticality perception. This additional analysis suggests that a CI model can also account for this latter measure of body orientation. In addition, we observed a significant interaction of the factor *group* and the linear term of the polynomial (F = 6.260, p = 0.012). This suggests a different effect of the visual stimulus in the two subgroups, more precisely, that the maximum bias was reached at higher visual tilt angles for the group of older participants. This could mean that aging might not only be associated with decreased sensory acuity, but also with changes in the tolerance for discrepancies: due to increased noise in the vestibular system with age, intersensory mismatches might be experienced more often during everyday life and thus be judged less unusual. As a prior, this might cause the brain to accept higher distortions in sensory information before inferring non-common causes of input. While Ramkhalawansingh et al. [28] argued that including available noisy information would increase older individuals’ risk of accidents in everyday life, the assumption of an increased tolerance in older participants is in line with observations described in a systematic review on multisensory integration by de Dieuleveult et al. [32]. The authors concluded that older participants tend to integrate all available information without weighting it according to its relevance or reliability. With respect to the model, this means that the segregation process might become apparent only for larger discrepancies for older compared to younger participants. The results of our experiment are consistent with this interpretation, as for older participants the maximum bias predicted from our data in the described model occurs at a larger visual tilt angle at ±31.12°, while for younger participants the maximum bias is reached at ±26.66°of visual tilt.

In conclusion, our data clearly revealed that the brain selectively reweights information on verticality provided by the different sub-systems during upright determination in situations of inter-sensory discrepancies. This process changes with age as the perception of own upright body orientation seems to be affected by a reduced reliability of the contributing sensory modalities as well as altered weights given to their inputs. As a consequence, older people are more susceptible to visual distortions than younger people.

## Supporting information

S1 Fig Individual raw data of participants’ SPV estimates. The amount of visual tilt is presented on the x-axis, participants responses on the y-axis. Green color represents the data of younger participants, data of older participants is presented in yellow. Positive values indicate clockwise tilt of camera and platform. Note that clockwise camera tilt translates to a counterclockwise percept of scene tilt.
(TIFF)

S1 File (DOCX)

S1 Data (CSV)
