# Supplementary figures and images for: Changes in the perception of upright body orientation with age
Source: PLoS One. 2020 May 29;15(5):e0233160. doi: 10.1371/journal.pone.0233160 (PMC7259641; doi:10.1371/journal.pone.0233160)

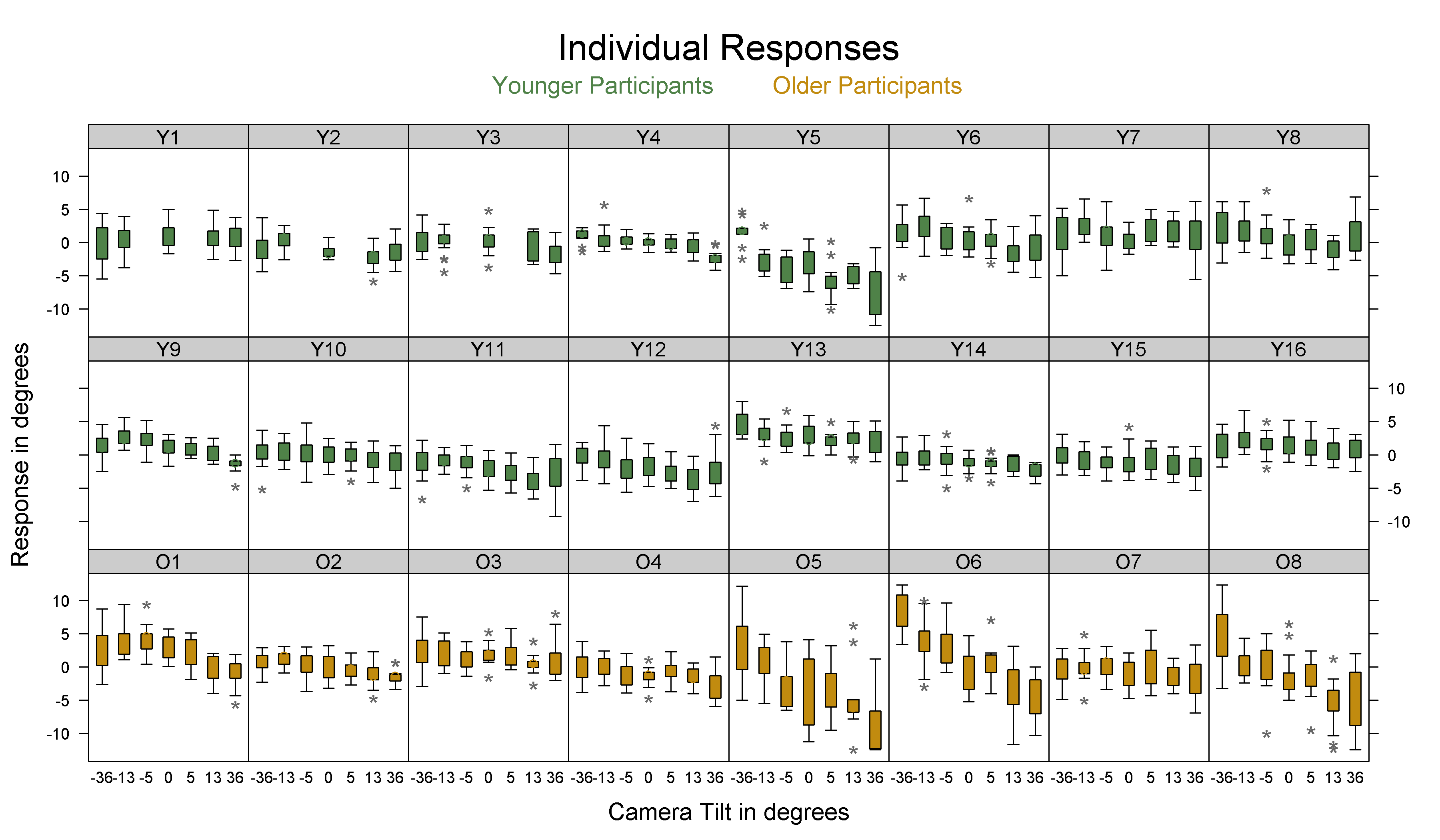

Supplement: S1 Fig — The amount of visual tilt is presented on the x-axis, participants responses on the y-axis. Green color represents the data of younger participants, data of older participants is presented in yellow. Positive values indicate clockwise tilt of camera and platform. Note that clockwise camera tilt translates to a counterclockwise percept of scene tilt. (TIFF) [file pone.0233160.s001.tiff]
