# Supplementary material for: Changes in the perception of upright body orientation with age
Source: PLoS One. 2020 May 29;15(5):e0233160. doi: 10.1371/journal.pone.0233160 (PMC7259641; doi:10.1371/journal.pone.0233160)
Supplement: S1 File — (DOCX) [file pone.0233160.s002.docx]

**Changes in the perception of upright body orientation with age**

**Supplementary material**

Sophia Nestmann ^1^, Hans-Otto Karnath* ^1,2^, Heinrich H. Bülthoff ^3^, Ksander Nikolas de Winkel ^3^

^1^Centre of Neurology, Division of Neuropsychology, Hertie-Institute for Clinical Brain Research, University of Tübingen, Tübingen, Germany

^2^ Department of Psychology, University of South Carolina, Columbia, USA

^3^ Max Planck Institute for Biological Cybernetics, Tübingen, Germany

As it not only seemed promising to interpret our data in a categorical manner, but also in terms of the Causal Inference model on verticality perception proposed by de Winkel et al. [1], we performed an additional analysis. This aimed at assessing whether the CI model is applicable to our data and to test the effect of age on the relationship between tilted visual input (​S​_V_) and SPV estimation from this perspective. According to the CI model, percepts will either result from *integration* or *segregation* of multisensory observations (signals). When observations are similar, they will be attributed to a common cause and integrated to form a percept. This means that the percept will be formed as a weighted sum of the observations. When observations are dissimilar, they will be attributed to independent causes, and observations deemed uninformative for a particular task will be discarded. Consequently, percepts form a mixture distribution with different components resulting from inferred causality.

Ideally, we would fit the CI model directly to the data and evaluate how the models’ parameter estimates change depending on the age of the participants. For the present experiment, this is however not straightforward: psychophysical experiments are generally designed in such a way that effects of manipulating stimuli S on responses R are measured; under the assumption that percepts P equal the responses, it is then possible to define a function f that describes the relation between stimuli and perception: R=P=f(S). However, in the present experiment the response did not equal the percept. Instead, for each experimental trial, the ‘response’ was a choice for inertial stimulus S_I_ that, combined with the experimentally predefined visual stimulus S_V,_ resulted in a percept P that subjectively equalled upright (0°): R=S_I_ for which P=f(S_V_,S_I_)=0. In the CI model, the expression relating stimuli to percepts f(S_I_,S_V_) is non-linear, and therefore the problem of solving it becomes intractable.

To nevertheless evaluate whether the CI model could also account for responses on the SPV task as a more direct evaluation of the subjective vertical than the Subjective Haptic Vertical (SHV) task used by de Winkel et al. [1], and to evaluate our hypothesis on an aging effect, we chose to approximate the behaviour of the CI-model instead: For the range of combinations of perceptually similar visual and inertial stimuli, the percept is constructed as a weighted average [2–4]. This implies that for a range of S​_V_, the ​S​_I_ that combines with it to result in a percept of 0° would follow a straight line with a negative slope, such as in Fig 1A).

For dissimilar combinations of visual and inertial stimuli, the CI model predicts that visual and inertial observations will be segregated, and that one of them will be discarded in the construction of a percept. We assume that it is the visual observation that would be discarded, as participants were aware that the visual stimulus could ‘fool’ them. This means that the percept of verticality would be based only on S_I_, and S_V_ is irrelevant. Consequently, the S_I_ that result in a percept of 0° for a range of S_V_ is simply a flat line (Fig 1B).

The CI model combines both possibilities, stating that the final percept is a weighted average of integration and segregation, where the weight depends on the magnitude of the discrepancy between S_V_ and S_I_. For the limited range of stimuli tested in the present experiment, this combination thus results in a sine-like relation between S_V_ and S_I_ (Fig 1C).


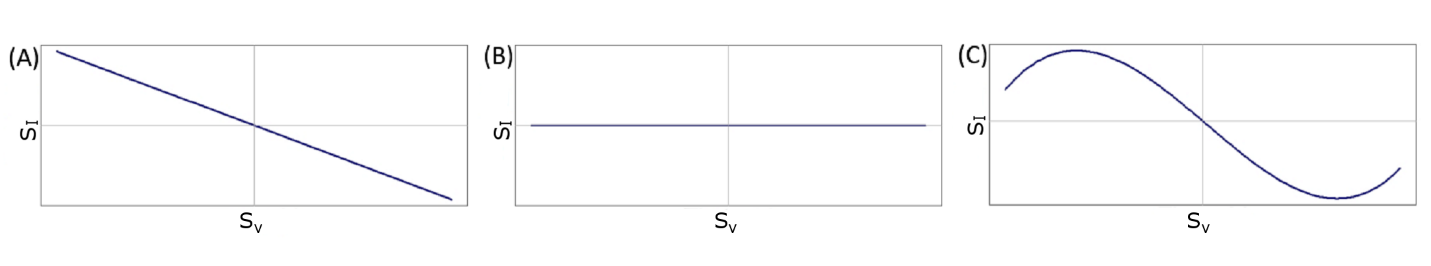


**Fig 1. Combinations of visual and inertial stimuli according to the CI model.**

(A) For small discrepancies between visual and inertial stimuli the CI model predicts that the percept is constructed as a weighted average of ​S​_V_ and S​_I_. For combinations that result in a percept of 0° this would be represented by a straight line with a negative slope. (B) For large discrepancies, the CI model predicts that visual and inertial observations will be segregated. We assume that the visual observation would be discarded, so that the percept of verticality would be based on S_I_ only. In this case, the S_I_ that results in a percept of 0° for a range of S_V_ is simply a flat line. (C) The CI model combines both possibilities, stating that the final percept is a weighted average of integration and segregation, where the weight depends on the magnitude of the discrepancy between S_V_ and S_I_. For the limited range of stimuli tested in the present experiment, this combination thus results in a sine-like relation between S_V_ and S_I_.

We approximated this behaviour by fitting a polynomial with a first degree and a third degree term, in the form of

$\text{SPV Estimate = }\beta_{1}*S_{v}+\beta_{2}*{S_{v}}^{3}$.

We only included odd terms, as we expected the responses to be symmetric around the origin. As a sanity check, we tested whether the chosen polynomial indeed provided the best approximation of the data by fitting different polynomial models of increasing complexity to our complete data set: we fitted polynomials of degree one, three and five to our data. The upper bound of five was chosen, since we have measured participants’ response at n=7 different tilt angles. In general, for seven points the highest degree polynomial, with thus the least residual variance, that can be fitted unambiguously to the data cloud is of degree six (n-1). However, as we only included odd terms, the upper bound was set to degree five. As we assumed the overall relationship to be the same for both groups, the model was fitted to the entire dataset, using maximum likelihood estimation. Model fits were evaluated using Akaike’s Information Criterion (AIC) and the Bayesian Information Criterion (BIC), which are based on the model likelihood and take into account the number of free parameters to prevent overfitting.

Table 1 shows the results of this analysis. As indicated by minima in both AIC and BIC scores, a polynomial including first and third degree coefficients describes the relationship of visual tilt and SPV estimation best. This interpretation is supported by likelihood ratio (LR) tests on the different models: An improved model fit is only observed for additionally including a cubic coefficient (*χ*^2^ (11) = 40.47, *p* < 0.001). A term of degree five did not increase the model fit any further. Based on these statistics, the polynomial including a first and third degree term was selected for the subsequent analysis. Residual plots for the model can be inspected in Figure 2.

**Table 1. Fit of model with increasing degree of the polynomial to the data.**

| **Degree of polynomial** | **df** | **AIC** | **BIC** | **logLik** | **χ2** | **p** |
| --- | --- | --- | --- | --- | --- | --- |
| 1 | 6 | 11419.44 | 11454.41 | - 5703.72 |  |  |
| 3 | 10 | 11373.31 | 11431.59 | - 5676.66 | 54.13 | < 0.001 |
| 5 | 15 | 11379.94 | 11467.36 | - 5674.97 | 3.37 | 0.643 |

Different models with increasing degree of the polynomial were fit to the complete dataset, not separating for different subgroups. Akaike information criterion (AIC) and Bayesian information criterion (BIC) were used to evaluate the model fit. Likelihood ratio tests were conducted to compare the estimated models. Log-likelihood ratio statistics, *χ*² values, degrees of freedom (df) as well as p-values of the analysis are presented.

**Figure 2. Residual plots of the model fit of a third-order polynomial to the complete data set.**

**
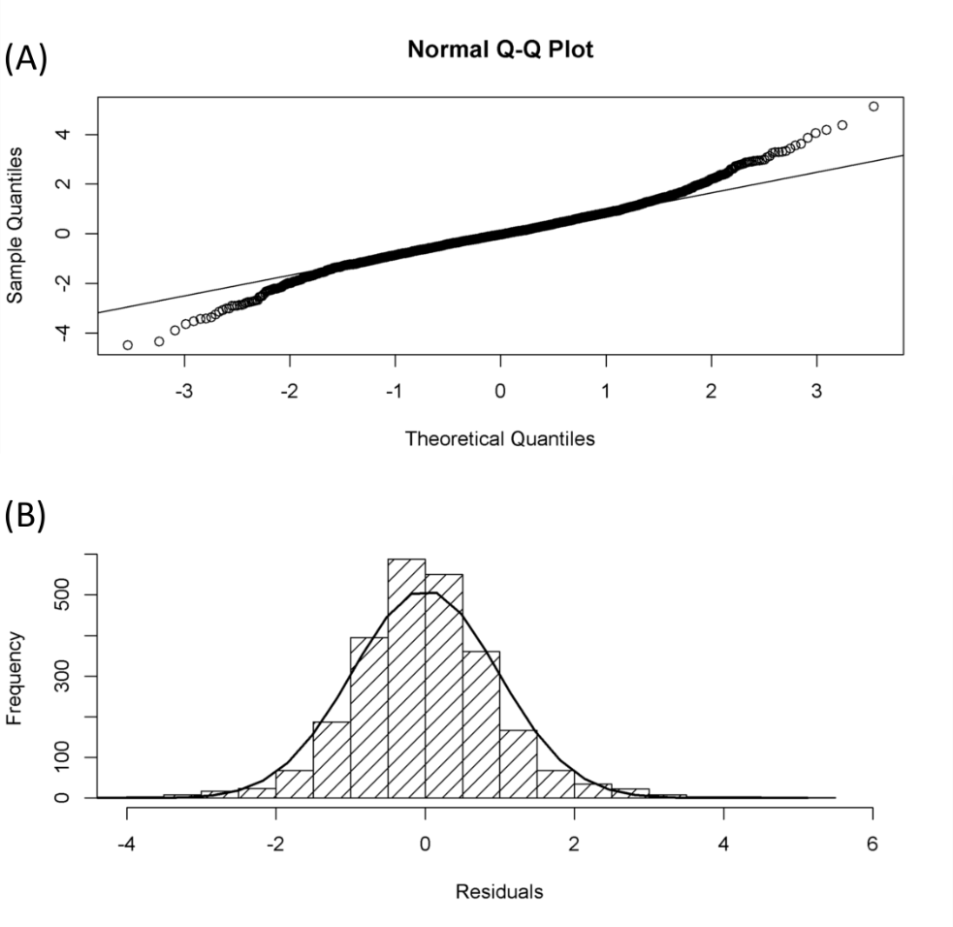
**(A) QQ-Plot comparing the expected residuals according to a normal distribution and the observed residuals. Visual inspection indicates a heavy tailed but symmetric distribution. Nevertheless, this will not lead to invalid estimations of model parameters [5] but might cause the test for the fixed effect to be more conservative [6]. (B) Histogram of observed residuals; added the expected normal distribution. Overall, visual inspection of the residual plots indicates a reasonably normal distribution.

To assess the relationship between visual input manipulation and the observed bias in SPV and to account for potential differences between groups, we fitted mixed effect models to the data using the lme function provided by R’s nlme package[7,8]. Random intercepts and slopes were estimated for the variable *participant*, to account for repeated measurements. A random intercept was estimated for the variable S_I_ to consider the potential effect of different starting angles of the platform. The above described polynomial as well as the factor *group* were included as fixed effects. Furthermore, we included the interaction of *​*S_V_ (polynomial) and *group* into our model. Graphically, an interaction effect would translate into a change of amplitude and thus an increased or decreased effect of visual tilt on the bias observed in SPV estimates. Heteroscedasticity was accounted for by allowing different variances in the subgroups, identified by the varIdent function provided by R’s nlme package[7,8]. The model was fit using restricted maximum likelihood (REML) estimation. Statistical significance of parameter estimates was determined using the anova function provided by R’s nlme package[7,8].

**Results.**

Results of the analysis indicate a main effect of the first degree (*F*= 19.590, *p*<0.001) and the third degree (*F*= 23.602, *p*<0.001) term of the polynomial describing the effect of *​*S_V_ on SPV estimates. Furthermore, we found a significant interaction of *group* and the first degree term of the polynomial (*F*=6.260, *p*= 0.012). No main effect of the factor *group* (F= 0.389, p=0.682) was observed (Table 2). Parameter estimates with the group of young participants set as reference group are summarized in Table 3. Coefficients indicate SPV bias in the direction of scene tilt. The absence of a main-effect for *group* indicates that there was no group-specific offset at S_V_ = 0. The interaction between the linear term and group indicates that the maximum bias is reached at higher angles of S​_V_ in the group of older participants.

**Table 2. Effect of age on the observed SPV bias.**

| **Model** | **Num df** | **Den df** | **F** | **P** |
| --- | --- | --- | --- | --- |
| Linear term of ​S_​V_ | 1 | 2411 | 19.590 | < 0.001 |
| Cubic Term of ​S_V_ | 1 | 2411 | 23.602 | < 0.001 |
| Group | 2 | 22 | 0.389 | 0.682 |
| Linear term of ​S_V_ x Group | 1 | 2411 | 6.260 | 0.012 |
| Cubic term of ​S_V_ x Group | 1 | 2411 | 0.833 | 0.361 |

The different components of the model are displayed together with the respective nominator degrees of freedom (Num df), denominator degrees of freedom (Den df), as well as *F*- and *p*-values of the analysis. Statistical significance of parameter estimates was determined using the anova function provided by R’s nlme package[7,8].

**Table 3. Parameter estimates of the model evaluation**

| **Model Component** | **ß** | **SD** | **df** | **t** | **p** |
| --- | --- | --- | --- | --- | --- |
| Linear term | -0.06973 | 0.01729 | 2411 | - 4.032 | < 0.001 |
| Cubic term | 0.00003 | 0.00001 | 2411 | 3.703 | < 0.001 |
| Younger Participants | -0.02100 | 0.15325 | 22 | -0.137 | 0.892 |
| Older Participants | 0.36154 | 0.22789 | 22 | 1.586 | 0.127 |
| Linear term x Older Participants | -0.07194 | 0.03184 | 2411 | - 2.260 | 0.024 |
| Cubic term x Older Participants | 0.00002 | 0.00002 | 2411 | 0.913 | 0.361 |

The table displays estimated coefficients for each model term, the corresponding standard deviations (SD), degrees of freedom (df), as well as t-statistics and p-values for each term. For interpretation of the coefficients, note that clockwise camera tilt translates into counterclockwise perception of visual tilt and thus negative weights indicate tilt in scene direction.

Model predictions were performed separately for younger and older participants by estimating separate mixed effect models. The centered data (see section ‘Data analysis’ of the main text) of the two groups as well as model predictions for this analysis can be inspected in Fig 3. Coefficients of a separate prediction for the polynomial of each subgroup as well as its predicted maxima and minima can be found in Table 4. We determined confidence intervals for the estimated maximum bias using a bootstrapping method. We split the data collected for older participants as well as for younger participants in halves. More precisely, we considered all possible combinations of participants for the subgroup of older participants (n=70) and 1000 out of all possible combinations for the subgroup of younger participants. For these different subsets we fitted the models and estimated the maximum bias. These estimates were averaged and standard deviations were calculated in order to determine the confidence intervals. These analyses show a larger bias in the subgroup of older participants (±2.93 ± 0.86°), for a larger visual tilt angle (±31.12°), compared to the subgroup of younger participants (±1.234± 0.19°, at ±26.66°).


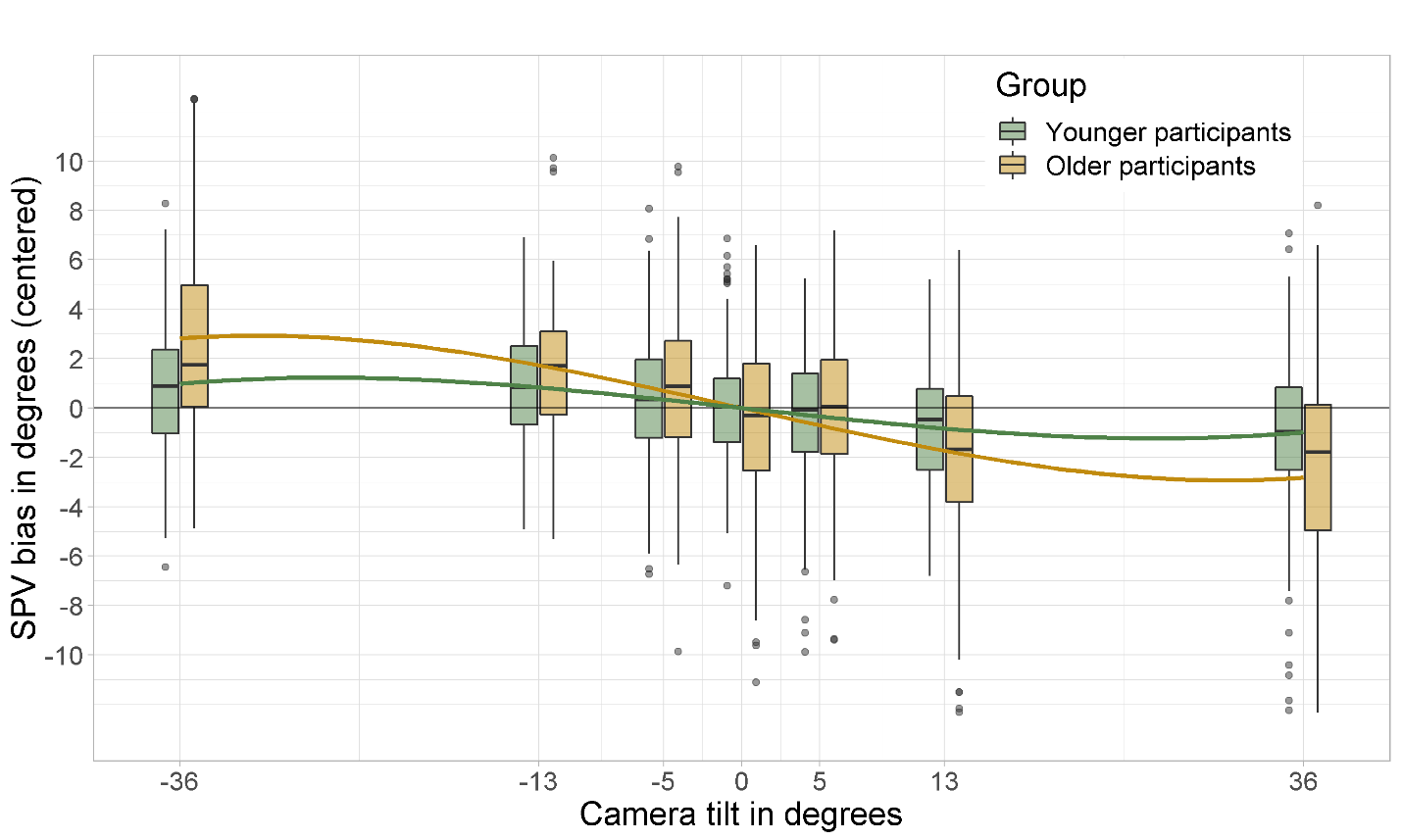


**Fig 3. Centered data and model predictions.**

Green colour represents the data (boxplots) and model predictions (polynomial) of young participants. Yellow colour represents the data and predictions for older participants. Positive values indicate clockwise tilt of camera and platform. Note that clockwise camera tilt translates to a counterclockwise percept of scene tilt. The bias in subjective postural vertical (SPV), when exposed to a tilted visual scene, was found to occur in the direction of scene tilt, stronger for older participants.

**Table 4. Separate model estimates for each group.**

| **Group** | **ß_1_** | **ß_2_** | **Maximum** | | | |
| --- | --- | --- | --- | --- | --- | --- |
|  |  |  | **Counterclockwise** | | **Clockwise** | |
|  |  |  | ***​*S_V_** | **SPV** | ***​*S_V_** | **SPV** |
| Younger Participants | -0.06921 | -0.00003 | -26.66° | 1.23° | 26.66 ° | -1.23° |
| Older Participants | -0.14126 | -0.00005 | -31.12° | 2.93° | 31.12° | -2.93° |

*ß*-weights represent the weight given to the different polynomial terms. Furthermore, maxima of biases in the estimation of subjective postural vertical (SPV) for each group both in clockwise and counterclockwise direction are presented together with the respective visual tilt angles (S_V_) at which they were predicted.

1. de Winkel KN, Katliar M, Diers D, Bülthoff HH. Causal Inference in the Perception of Verticality. Sci Rep. 2018 Apr 3;8(1):5483.

2. Ernst MO, Banks MS. Humans integrate visual and haptic information in a statistically optimal fashion. Nature. 2002 Jan 24;415(6870):429–33.

3. Hillis JM, Ernst MO, Banks MS, Landy MS. Combining sensory information: mandatory fusion within, but not between, senses. Science. 2002 Nov 22;298(5598):1627–30.

4. Ernst MO, Bülthoff HH. Merging the senses into a robust percept. Trends Cogn Sci. 2004 Apr;8(4):162–9.

5. Jacqmin-Gadda H, Sibillot S, Proust C, Molina J-M, Thiébaut R. Robustness of the linear mixed model to misspecified error distribution. Comput Stat Data Anal. 2007 Jun;51(10):5142–54.

6. Pinheiro JC, Bates DM. Mixed-effects models in S and S-PLUS. reprinted paperback ed. of the 2000 ed. New York: Springer; 2009. 528 p. (Statistics and computing).

7. Pinheiro J, Bates D, DebRoy S, Sarkar D, R Core Team. nlme: Linear and Nonlinear Mixed Effects Models [Internet]. 2018. Available from: https://CRAN.R-project.org/package=nlme

8. R Development Core Team. R: A Language and Environment for Statistical Computing [Internet]. Vienna, Austria: R Foundation for Statistical Computing; 2018. Available from: http://www.R-project.org
